# Socio-demographic, clinical and service use determinants associated with HIV related stigma among people living with HIV/AIDS: a systematic review and meta-analysis

**DOI:** 10.1186/s12913-021-06980-6

**Published:** 2021-09-22

**Authors:** Bahram Armoon, Peter Higgs, Marie-Josée Fleury, Amir-Hossien Bayat, Ladan Fattah Moghaddam, Azadeh Bayani, Yadollah Fakhri

**Affiliations:** 1 Research Center, Douglas Mental Health University Institute, 6875 LaSalle Blvd, Montreal, QC H4H 1R3 Canada; 2 Department of Psychiatry, McGill University, 1033, Pine Avenue West, Montreal, QC H3A 1A1 Canada; 3 Department of Public Health, School of Psychology and Public Health, La Trobe University, Melbourne, VIC Australia; 4 Management, Evaluation and Health Policies Department, School of Public Health, Université de Montréal, 7101 av. du Parc, Montreal, QC H3X1X9 Canada; 5 Social Determinants of Health Research Center, Saveh University of Medical Sciences, Saveh, Iran; 6 Department of nursing, faculty of nursing and midwifery, Tehran medical sciences, Islamic Azad University, Tehran, Iran; 7 Student Research Committee, School of Allied Medical Sciences, Shahid Beheshti University of Medical Sciences, Tehran, Iran; 8 Social Determinants in Health Promotion Research Center, Hormozgan Health Institute, Hormozgan University of Medical Sciences, Bandar Abbas, Iran

**Keywords:** HIV-related stigma, CD4 count, Medication adherence, Time since diagnosis

## Abstract

**Background:**

Defining HIV-related stigma (HRS) can be problematic due to structural inequalities, cultural differences, discrimination by health care providers and the limitations of tools measuring stigma for people living with HIV (PLWH). This meta-analysis aimed to determine self-reported HRS and its association with socio-demographic and clinical determinants.

**Methods:**

PubMed, Scopus, Web of Science, PsycInfo, SciELO and Cochrane electronic databases were searched and after reviewing for study duplicates, the full-text of selected articles were assessed for eligibility using Population, Intervention, Comparator, Outcomes criteria. We used fixed and random-effects meta-analysis models to estimate the pooled prevalence, pooled odds ratio (OR) and 95% confidence intervals.

**Results:**

Thirty-one studies containing 10,475 participants met the eligibility criteria. Among the potential risk factors: age > 30 years (OR = 0.93, 95%CI = 0.86, 1), living with a spouse (OR = 0.07, 95%CI = 0.02, 0.17), CD4 count < 200 (OR = 0.5, 95% CI = 0.31, 0.68), medication adherence (OR = 0.96, 95%CI = 0.94, 0.99), poor access to care (OR = 0.79, 95%CI = 0.65, 0.93), time since diagnosis, and accessibility to care (OR = 0.37, 95%CI = 0.11, 0.86) were all significantly associated with self-reported HIV stigma among PWLH.

**Conclusion:**

Stigma is correlated with numerous negative consequences in marginalised populations including PLWH. Considering the negative association that stigma has on HIV prevention and treatment targeted evidence-based stigma reduction interventions are recommended. Interventions that are focused on a particular group, such as healthcare professionals are warranted. Rigorously designed studies with specific and validated outcome measures associated with targeted interventions may help to improve the reduction of HRS for PLWH.

## Background

Stigma is a social phenomenon known to have a negative impact on the lives of people living with HIV (PLWH) [1]. However, defining HIV-related stigma (HRS) is difficult because of the intersection it has with structural inequalities, cultural differences, discrimination by health care providers and also the limitations with the tools that measure stigma among PLWH [2–5]. While previous research has examined the association between stigma and HIV treatment, including access to HIV care [6, 7], stigma is also considered as a key barrier to HIV antiretroviral therapy (ART) adherence [8]. High rates of stigma have been reported for PLWH who have limited access to ART [9]. Despite previous research suggesting access to ART services can protect against HRS [10], sustained adherence remains crucial in HIV-related care [11]. The effectiveness of HIV prevention and care services are affected by HRS with previous studies describing significant barriers to voluntary testing and counseling among PLWH [12–14]. HRS is associated with difficulties accessing care due to discrimination and fear of rejection from health services [15]. The high levels of stigma experienced by PLWH also affect mental health conditions such as depression [15].

Our study differs from recent research [6, 16] as it systematically reviews what has been completed so far and applied odds ratios to determine relationships between socio-demographic, clinical and service use determinants and HRS among PLWH. Rrueda et al. [6] investigated the association of variables such as mental health, physical health, quality of life and medication adherence with HRS and Klein et al. [16] considered interventions to prevent HIV-related stigma and discrimination but did not consider the association between any variables with HRS. To the best of our knowledge no study has considered the demographic and clinical variables such as CD4 < 200 and other service use variables such as poor access to care, and the impact that time since diagnosis has on care.

Importantly, we not focus only on one variable but include any variable in the meta-analysis. One of the challenges is the small number of high quality studies describing the correlation between socio-demographic, clinical and service use determinants, and insufficient evidence for HIV/AIDS prevention and control programs to inform specific tailored intervention strategies. To understand the factors affecting HRS, this study considered the following outcomes: 1) socio-demographic (age > 30 years, being female, illiterate and primary education, <$USD100 monthly income, living with a spouse), 2) clinical factors (CD4 count < 200), and 3) service use (previous HIV testing, medication adherence, poor access to care, time since diagnosis and accessibility to care) determinants for HRS among PWLH.

This study is a meta-analysis of peer reviewed journal articles published before November 1, 2020 assessing the correlation between socio-demographic, clinical and service use determinants and HRS among PLWH.

## Methods

### Search strategy and study selection

Our study was implemented using the Protocols of Systematic Reviews and Meta-Analyses (PRISMA) guidelines [17–20].

According to the search strategy and additional manual searches from the article references, 10,457 articles from four databases were found. For the article inclusion, two independent researchers (AB and BA) reviewed the electronic databases of PubMed, Scopus, Web of science, PsycInfo, SciELO and Cochrane electronic database, with the following search keywords: (Social Determinants of Health [MeSH Terms]) or (Socioeconomic Factors [MeSH Terms]) or (Spouses [MeSH Terms]) or (Literacy [MeSH Terms]) or (Medication Adherence [MeSH Terms]) or (CD4 Lymphocyte Count [MeSH Terms]) or (Health Services Accessibility [MeSH Terms]) or (Time-to-Treatment [MeSH Terms]) or (Time to diagnosis [Title/Abstract]) or (previous testing [Title/Abstract]) and (HIV [MeSH Terms]) and (social stigma [MeSH Terms]) or stigma [Title/Abstract]) or (shame [MeSH Terms]) or (Self Disclosure [MeSH Terms]) or (Self Concept [MeSH Terms]) or (Negative Self-Image [Title/Abstract]) or (blame [Title/Abstract]) or (feel guilty [Title/Abstract]) and (people who lived with HIV [Title/Abstract]) or (living with HIV [Title/Abstract]).

We show the search strategy in Table 1. The references were managed by EndNote X7 software (Thomson Reuters). Duplicate articles were excluded. Initially, two researchers reviewed the extracted article titles and abstracts independently, based on population, intervention, comparison, outcome (PICO) criteria. A third (AMB) member of the research team provided the required input, and resolved disagreements about articles included in the study. Secondly, AB and BA assessed the full articles, considering the study inclusion criteria based on PICO, and exclusion criteria including having no access to the full article and manuscripts missing principal data. Only articles written in English were included.

**Table 1:** search strategy

*Search number*	*Query*	*Item founds*
*PubMed Search*		
26	(((((((Social Determinants of Health [MeSH Terms]) OR (Socioeconomic Factors [MeSH Terms])) OR (Spouses [MeSH Terms])) OR (Literacy [MeSH Terms])) OR ((((((Medication Adherence [MeSH Terms]) OR (CD4 Lymphocyte Count [MeSH Terms])) OR (Health Services Accessibility [MeSH Terms])) OR (Time-to-Treatment [MeSH Terms])) OR (Time to diagnosis [Title/Abstract])) OR (previous testing [Title/Abstract]))) AND (HIV [MeSH Terms])) AND ((((((social stigma [MeSH Terms]) OR stigma [Title/Abstract]) OR (shame [MeSH Terms])) OR (Self Disclosure [MeSH Terms])) OR (Self Concept [MeSH Terms])) OR (Negative Self-Image [Title/Abstract])) OR (blame [Title/Abstract])) OR (feel guilty [Title/Abstract]))) AND ((people who lived with HIV [Title/Abstract]) OR (living with hiv [Title/Abstract]))	
25	HIV [MeSH Terms]	
24	(people who lived with HIV [Title/Abstract]) OR (living with hiv [Title/Abstract])	
23	((((((stigma [Title/Abstract]) OR (shame [MeSH Terms])) OR (Self Disclosure [MeSH Terms])) OR (Self Concept [MeSH Terms])) OR (Negative Self-Image [Title/Abstract])) OR (blame [Title/Abstract])) OR (feel guilty [Title/Abstract])	
22	(((((Medication Adherence [MeSH Terms]) OR (CD4 Lymphocyte Count [MeSH Terms])) OR (Health Services Accessibility [MeSH Terms])) OR (Time-to-Treatment [MeSH Terms])) OR (Time to diagnosis [Title/Abstract])) OR (previous testing [Title/Abstract])	
21	(((Social Determinants of Health [MeSH Terms]) OR (Socioeconomic Factors [MeSH Terms])) OR (Spouses [MeSH Terms])) OR (Literacy [MeSH Terms])	
20	living with hiv [Title/Abstract]	
19	people who lived with HIV [Title/Abstract]	
18	feel guilty [Title/Abstract]	
17	blame [Title/Abstract]	
16	Negative Self-Image [Title/Abstract]	
15	Self Concept [MeSH Terms]	
14	Self Disclosure [MeSH Terms]	
13	shame [MeSH Terms]	
12	stigma [Title/Abstract]	
11	Social Stigma [MeSH Terms]	
10	previous testing [Title/Abstract]	
9	Time to diagnosis [Title/Abstract]	
8	Time-to-Treatment [MeSH Terms]	
7	Health Services Accessibility [MeSH Terms]	
6	CD4 Lymphocyte Count [MeSH Terms]	
5	Medication Adherence [MeSH Terms]	
4	Literacy [MeSH Terms]	
3	Spouses [MeSH Terms]	
2	Socioeconomic Factors [MeSH Terms]	
1	Social Determinants of Health [MeSH Terms]	
Cochrane search		
*ID*	*Search*	
#1	MeSH descriptor: [HIV] explode all trees	
#2	MeSH descriptor: [Social Stigma] explode all trees	
#3	(stigma):ti (Word variations have been searched)	
#4	(people who lived with HIV):ti (Word variations have been searched)	
#5	(Living with HIV):ti (Word variations have been searched)	
#6	MeSH descriptor: [Shame] explode all trees	
#7	MeSH descriptor: [Self Disclosure] explode all trees	
#8	MeSH descriptor: [Self Concept] explode all trees	
#9	(Negative Self-Image):ti (Word variations have been searched)	
#10	(blame):ti (Word variations have been searched)	
#11	(feel guilty):ti (Word variations have been searched)	
#12	MeSH descriptor: [Social Determinants of Health] explode all trees	
#13	MeSH descriptor: [Socioeconomic Factors] explode all trees	
#14	MeSH descriptor: [Spouses] explode all trees	
#15	MeSH descriptor: [Literacy] explode all trees	
#16	MeSH descriptor: [Medication Adherence] explode all trees	
#17	MeSH descriptor: [CD4 Lymphocyte Count] explode all trees	
#18	MeSH descriptor: [Health Services Accessibility] explode all trees	
#19	MeSH descriptor: [Time-to-Treatment] explode all trees	
#20	(Time to diagnosis):ti (Word variations have been searched)	
#21	(previous testing):ti (Word variations have been searched)	
#22	#12 OR #13 OR #14 OR #15 OR #16 OR #17 OR #18 OR #19 OR #20	
#23	#2 OR #3 OR #6 OR #7 OR #8 OR #9 OR #10 OR #11	
#24	#4 OR #5	
#25	#22 AND #1 AND #23 AND #24	
#26	#22 AND #23 AND #24	
*Web of knowledge*		
*#24*	*#21 AND #22 AND #23* *Indexes = SCI-EXPANDED, SSCI, A&HCI, CPCI-S, CPCI-SSH, BKCI-S, BKCI-SSH, ESCI, CCR-EXPANDED, IC Timespan = All years*	
*#23*	*#19 OR #20* *Indexes = SCI-EXPANDED, SSCI, A&HCI, CPCI-S, CPCI-SSH, BKCI-S, BKCI-SSH, ESCI, CCR-EXPANDED, IC Timespan = All years*	
*#22*	*#11 OR #12 OR #13 OR #14 OR #15 OR #16 OR #17 OR #18* *Indexes = SCI-EXPANDED, SSCI, A&HCI, CPCI-S, CPCI-SSH, BKCI-S, BKCI-SSH, ESCI, CCR-EXPANDED, IC Timespan = All years*	
*#21*	*#1 OR #2 OR #3 OR #4 OR #5 OR #6 OR #7 OR #8 OR #9 OR #10* *Indexes = SCI-EXPANDED, SSCI, A&HCI, CPCI-S, CPCI-SSH, BKCI-S, BKCI-SSH, ESCI, CCR-EXPANDED, IC Timespan = All years*	
*#20*	*TI = (Living with HIV)* *Indexes = SCI-EXPANDED, SSCI, A&HCI, CPCI-S, CPCI-SSH, BKCI-S, BKCI-SSH, ESCI, CCR-EXPANDED, IC Timespan = All years*	
*#19*	*TI = (people who lived with HIV)* *Indexes = SCI-EXPANDED, SSCI, A&HCI, CPCI-S, CPCI-SSH, BKCI-S, BKCI-SSH, ESCI, CCR-EXPANDED, IC Timespan = All years*	
*#18*	*TI = (Shame)* *Indexes = SCI-EXPANDED, SSCI, A&HCI, CPCI-S, CPCI-SSH, BKCI-S, BKCI-SSH, ESCI, CCR-EXPANDED, IC Timespan = All years*	
*#17*	*TI = (feel guilty)* *Indexes = SCI-EXPANDED, SSCI, A&HCI, CPCI-S, CPCI-SSH, BKCI-S, BKCI-SSH, ESCI, CCR-EXPANDED, IC Timespan = All years*	
*#16*	*TI = (blame)* *Indexes = SCI-EXPANDED, SSCI, A&HCI, CPCI-S, CPCI-SSH, BKCI-S, BKCI-SSH, ESCI, CCR-EXPANDED, IC Timespan = All years*	
*#15*	*TI = (Negative Self-Image)* *Indexes = SCI-EXPANDED, SSCI, A&HCI, CPCI-S, CPCI-SSH, BKCI-S, BKCI-SSH, ESCI, CCR-EXPANDED, IC Timespan = All years*	
*#14*	*TI = (Self Concept)* *Indexes = SCI-EXPANDED, SSCI, A&HCI, CPCI-S, CPCI-SSH, BKCI-S, BKCI-SSH, ESCI, CCR-EXPANDED, IC Timespan = All years*	
*#13*	*TI = (Self Disclosure)* *Indexes = SCI-EXPANDED, SSCI, A&HCI, CPCI-S, CPCI-SSH, BKCI-S, BKCI-SSH, ESCI, CCR-EXPANDED, IC Timespan = All years*	
*#12*	*TI = (Stigma)* *Indexes = SCI-EXPANDED, SSCI, A&HCI, CPCI-S, CPCI-SSH, BKCI-S, BKCI-SSH, ESCI, CCR-EXPANDED, IC Timespan = All years*	
*#11*	*TI = (Social Stigma)* *Indexes = SCI-EXPANDED, SSCI, A&HCI, CPCI-S, CPCI-SSH, BKCI-S, BKCI-SSH, ESCI, CCR-EXPANDED, IC Timespan = All years*	
*#10*	*TI = (previous testing)* *Indexes = SCI-EXPANDED, SSCI, A&HCI, CPCI-S, CPCI-SSH, BKCI-S, BKCI-SSH, ESCI, CCR-EXPANDED, IC Timespan = All years*	
*#9*	*TI = (*Time to diagnosis*)* *Indexes = SCI-EXPANDED, SSCI, A&HCI, CPCI-S, CPCI-SSH, BKCI-S, BKCI-SSH, ESCI, CCR-EXPANDED, IC Timespan = All years*	
*#8*	*TI = (Time-to-Treatment)* *Indexes = SCI-EXPANDED, SSCI, A&HCI, CPCI-S, CPCI-SSH, BKCI-S, BKCI-SSH, ESCI, CCR-EXPANDED, IC Timespan = All years*	
*#7*	*TI = (Health Services Accessibility)* *Indexes = SCI-EXPANDED, SSCI, A&HCI, CPCI-S, CPCI-SSH, BKCI-S, BKCI-SSH, ESCI, CCR-EXPANDED, IC Timespan = All years*	
*#6*	*TI = (CD4 Lymphocyte Count)* *Indexes = SCI-EXPANDED, SSCI, A&HCI, CPCI-S, CPCI-SSH, BKCI-S, BKCI-SSH, ESCI, CCR-EXPANDED, IC Timespan = All years*	
*#5*	*TI = (Medication Adherence)* *Indexes = SCI-EXPANDED, SSCI, A&HCI, CPCI-S, CPCI-SSH, BKCI-S, BKCI-SSH, ESCI, CCR-EXPANDED, IC Timespan = All years*	
*#4*	*TI = (Literacy)* *Indexes = SCI-EXPANDED, SSCI, A&HCI, CPCI-S, CPCI-SSH, BKCI-S, BKCI-SSH, ESCI, CCR-EXPANDED, IC Timespan = All years*	
*#3*	*TI = (Spouses)* *Indexes = SCI-EXPANDED, SSCI, A&HCI, CPCI-S, CPCI-SSH, BKCI-S, BKCI-SSH, ESCI, CCR-EXPANDED, IC Timespan = All years*	
*#2*	*TI = (Socioeconomic Factors)* *Indexes = SCI-EXPANDED, SSCI, A&HCI, CPCI-S, CPCI-SSH, BKCI-S, BKCI-SSH, ESCI, CCR-EXPANDED, IC Timespan = All years*	
*#1*	*TI = (Social Determinants of Health)* *Indexes = SCI-EXPANDED, SSCI, A&HCI, CPCI-S, CPCI-SSH, BKCI-S, BKCI-SSH, ESCI, CCR-EXPANDED, IC Timespan = All years*	
*PsycINFO search*		
	*(“Social Determinants of Health” OR “Socioeconomic Factors OR Spouses OR” Literacy” OR” Medication Adherence” OR “CD4 Lymphocyte Count” OR “Health Services Accessibility OR Time-to-Treatment OR” Time to diagnosis” OR” previous testing”) AND (“Shame” OR “Self-Disclosure” OR “Self-Concept” OR “Negative Self-Image” OR “blame” OR “feel guilty”) AND (“people who lived with HIV” OR “Living with HIV”)*	
*Scielo search*		
	*Social Determinants of Health [Title words] or Socioeconomic Factors [Title words] or Spouses [Title words] or Literacy [Title words] or Medication Adherence [Title words] or CD4 Lymphocyte Count [Title words] or Health Services Accessibility [Title words] or Time-to-Treatment [Title words] or Time to diagnosis [Title words] or previous testing [Title words] and Shame [Title words] or Self-Disclosure [Title words] or Self-Concept [Title words] or Negative Self-Image [Title words] or blame [Title words] or feel guilty [Title words] and people who lived with HIV [Title words] or Living with HIV [Title words]*	
	Scopus search	Item found
1-	(((TITLE-ABS-KEY (social AND determinants AND of AND health)) OR (TITLE-ABS-KEY (socioeconomic AND factors)) OR (TITLE-ABS-KEY (spouses)) OR (TITLE-ABS-KEY (literacy))) OR ((TITLE-ABS-KEY (medication AND adherence)) OR (TITLE-ABS-KEY (cd4 AND lymphocyte AND count)) OR (TITLE-ABS-KEY (health AND services AND accessibility)) OR (TITLE-ABS-KEY (time-to-treatment)) OR (TITLE-ABS-KEY (time AND to AND diagnosis)) OR (TITLE-ABS-KEY (previous AND testing)))) AND (TITLE-ABS-KEY (hiv)) AND ((TITLE-ABS-KEY (stigma)) OR (TITLE-ABS-KEY (social AND stigma)) OR (TITLE-ABS-KEY (shame)) OR (TITLE-ABS-KEY (self AND disclosure)) OR (TITLE-ABS-KEY (self AND concept)) OR (TITLE-ABS-KEY (negative AND self-image)) OR (TITLE-ABS-KEY (blame)) OR (TITLE-ABS-KEY (feel AND guilty))) AND ((TITLE-ABS-KEY (people AND who AND lived AND with AND hiv)) OR (TITLE-ABS-KEY (living AND with AND hiv))) ...View More	
2-	((TITLE-ABS-KEY (social AND determinants AND of AND health)) OR (TITLE-ABS-KEY (socioeconomic AND factors)) OR (TITLE-ABS-KEY (spouses)) OR (TITLE-ABS-KEY (literacy))) OR ((TITLE-ABS-KEY (medication AND adherence)) OR (TITLE-ABS-KEY (cd4 AND lymphocyte AND count)) OR (TITLE-ABS-KEY (health AND services AND accessibility)) OR (TITLE-ABS-KEY (time-to-treatment)) OR (TITLE-ABS-KEY (time AND to AND diagnosis)) OR (TITLE-ABS-KEY (previous AND testing))) ...View More	
3-	(TITLE-ABS-KEY (people AND who AND lived AND with AND hiv)) OR (TITLE-ABS-KEY (living AND with AND hiv))	
4-	(TITLE-ABS-KEY (stigma)) OR (TITLE-ABS-KEY (social AND stigma)) OR (TITLE-ABS-KEY (shame)) OR (TITLE-ABS-KEY (self AND disclosure)) OR (TITLE-ABS-KEY (self AND concept)) OR (TITLE-ABS-KEY (negative AND self-image)) OR (TITLE-ABS-KEY (blame)) OR (TITLE-ABS-KEY (feel AND guilty))	
5-	(TITLE-ABS-KEY (medication AND adherence)) OR (TITLE-ABS-KEY (cd4 AND lymphocyte AND count)) OR (TITLE-ABS-KEY (health AND services AND accessibility)) OR (TITLE-ABS-KEY (time-to-treatment)) OR (TITLE-ABS-KEY (time AND to AND diagnosis)) OR (TITLE-ABS-KEY (previous AND testing))	
6-	(TITLE-ABS-KEY (social AND determinants AND of AND health)) OR (TITLE-ABS-KEY (socioeconomic AND factors)) OR (TITLE-ABS-KEY (spouses)) OR (TITLE-ABS-KEY (literacy))	
7-	TITLE-ABS-KEY (living AND with AND hiv)	
8-	TITLE-ABS-KEY (people AND who AND lived AND with AND hiv)	
9-	TITLE-ABS-KEY (feel AND guilty)	
10-	TITLE-ABS-KEY (blame)	
11-	TITLE-ABS-KEY (negative AND self-image)	
12-	TITLE-ABS-KEY (self AND concept)	
13-	TITLE-ABS-KEY (self AND disclosure)	
14-	TITLE-ABS-KEY (shame)	
15-	TITLE-ABS-KEY (social AND stigma)	
16-	TITLE-ABS-KEY (hiv)	
17-	TITLE-ABS-KEY (stigma)	
18-	TITLE-ABS-KEY (hiv)	
19-	TITLE-ABS-KEY (previous AND testing)	
20-	TITLE-ABS-KEY (time AND to AND diagnosis)	
21-	TITLE-ABS-KEY (time-to-treatment)	
22-	TITLE-ABS-KEY (health AND services AND accessibility)	
23-	TITLE-ABS-KEY (cd4 AND lymphocyte AND count)	
24-	TITLE-ABS-KEY (medication AND adherence)	
25-	TITLE-ABS-KEY (literacy)	
26-	TITLE-ABS-KEY (spouses)	
27-	TITLE-ABS-KEY (socioeconomic AND factors)	

### Inclusion criteria based on PICO

Population: people living with HIV.

Intervention: PLWH who report HRS.

The comparison group: PLWH not reporting HRS.

Outcomes: Positive and protective association of the social-demographic factors, clinical and service use determinant of PLWH on HRS.

We reviewed cross-sectional, cohort, and case-control studies. According to the PICO criteria, for the “population”, only PLWH were included; the “intervention” targeted HRS; the “comparison” group was PLWH not reporting HRS; “outcomes” were the significant association of the social-demographic factors, clinical and service use determinant of HRS on PLWH.; “study design” included cross-sectional, cohort or case-control studies. Qualitative studies, secondary studies not reporting primary data, systematic reviews and meta-analysis studies were excluded. Articles that had major heterogeneity or outcome variations from the study groups were excluded. Articles or variables that were not investigated extensively enough to be included in the meta-analysis were also not considered as associated variables of HRS among PLWH (i.e. opportunistic infection, quality of life, self-efficacy, helplessness previous testing, HIV knowledge, prison history).

### Instruments

Berger Stigma Scale, [21] HIV Stigma Measure, [22] Internalized AIDS-Related Stigma Scale, [23] and Demi-HIV Stigma Scale were the most frequently applied tools for measuring HIV-related stigma.

### Data extraction and study quality assessment

The quality of each paper was evaluated applying the Newcastle-Ottawa Scale [24] recommended by the Cochrane Collaboration [25] (See Table 2). Two researchers (AB and BA) evaluated the studies separately applying a standardized data collection form (excel form). Any contradictions of opinions about quality of overall studies between the authors were resolved by YF and AB through discussion. The surname of the first author, publication year, demographic data of participants (age > 30 years, living with spouse, education) and other features such as CD4 count < 200, previous HIV testing and service use determinants such as medication adherence, poor access to care, time since diagnosis and accessibility to care were recorded during the data extraction.

**Table 2:** Risk of bias assessment using Newcastle-Ottawa scale

Study	Selection (***)	Comparability (*)	Exposure/outcome (*)	Method of assessment	Quality Assessment	Quality Assessment score
Charles et al. [9]	***		*	Newcastle-Ottawa scale adapted for cross-sectional studies	Good	4
Waite et al. [26]	*	*	*	Newcastle-Ottawa scale adapted for cross-sectional studies	Satisfactory	3
Li et al. [27]	**	*	*	Newcastle-Ottawa scale adapted for cross-sectional studies	Good	4
Egbe et al. [28]	***		*	Newcastle-Ottawa scale adapted for cross-sectional studies	Good	4
Emlet et al. [29]	***	*		Newcastle-Ottawa scale adapted for cross-sectional studies	Good	4
Akena et al. [30]	*	*	*	Newcastle-Ottawa scale adapted for cross-sectional studies	Satisfactory	3
Letshwenyo-Maruatona et al. [31]	*	*	*	Newcastle-Ottawa scale adapted for cross-sectional studies	Satisfactory	3
Chan et al. [32]	**	*	*	Newcastle-Ottawa scale adapted for cross-sectional studies	Good	4
Wu et al. [33]	***	*	*	Newcastle-Ottawa scale adapted for cross-sectional studies	Very Good	5
Rael and Hampanda [34]	**	*	*	Newcastle-Ottawa scale adapted for cross-sectional studies	Good	4
Zhang et al. [35]	**	*	*	Newcastle-Ottawa scale adapted for cross-sectional studies	Good	4
Peltzer and Ramlagan [36]	***	*	*	Newcastle-Ottawa scale adapted for cross-sectional studies	Very Good	5
Zhang et al. [37]	**	*	*	Newcastle-Ottawa scale adapted for cross-sectional studies	Good	4
Srithanaviboonchai et al. [38]	**	*	*	Newcastle-Ottawa scale adapted for cross-sectional studies	Good	4
Li et al. [39]	**	*	*	Newcastle-Ottawa scale adapted for cross-sectional studies	Good	4
Stangl et al. [40]	***	*	*	Newcastle-Ottawa scale adapted for cross-sectional studies	Very Good	5
Stevelink et al. [41]	***	*	*	Newcastle-Ottawa scale adapted for cross-sectional studies	Very Good	5
Zhang et al. [42]	***	*	*	Newcastle-Ottawa scale adapted for cross-sectional studies	Very Good	5
Rivera et al. [43]	**	*	*	Newcastle-Ottawa scale adapted for cross-sectional studies	Good	4
Sayles et al. [15]	*	*	*	Newcastle-Ottawa scale adapted for cross-sectional studies	Satisfactory	3
Li et al. [44]	**	*	*	Newcastle-Ottawa scale adapted for cross-sectional studies	Good	4
Genberg et al. [45]	**	*	*	Newcastle-Ottawa scale adapted for cross-sectional studies	Good	4
Earnshaw et al. [46]	***	*	*	Newcastle-Ottawa scale adapted for cross-sectional studies	Very Good	5
Burke et al. [47]	***	*	*	Newcastle-Ottawa scale adapted for cross-sectional studies	Very Good	5
Turan et al. [48]	**	*	*	Newcastle-Ottawa scale adapted for cross-sectional studies	Good	4
Mao et al. [49]	**	*	*	Newcastle-Ottawa scale adapted for cross-sectional studies	Good	4
Zhang et al. [50]	*	*	*	Newcastle-Ottawa scale adapted for cross-sectional studies	Satisfactory	3
Rintamaki et al. [51]	**	*	*	Newcastle-Ottawa scale adapted for cross-sectional studies	Good	4
Liu et al. [52]	**	*	*	Newcastle-Ottawa scale adapted for cross-sectional studies	Good	4
Wolitski et al. [53]	**	*	*	Newcastle-Ottawa scale adapted for cross-sectional studies	Good	4
Levi-Minzi and Surratt [54]	***	*	*	Newcastle-Ottawa scale adapted for cross-sectional studies	Very Good	5

Cross-sectional studies:

* Unsatisfactory studies

*** Satisfactory studies

**** Good studies

***** Very good studies

### Data synthesis and statistical analysis

The meta-analysis was produced by pooling odds ratios (ORs) with 95% confidence intervals recognizing social and risk-taking behaviors related to HIV stigma among PLWH. We applied Q test with a *P* value < 0.05 and I^2^ statistics with a cutoff of ≥50% to evaluate the correlations across the studies. We also obtained uncertainty 95% confidence intervals for I^2^. We assumed any negative values to I2 as equal to zero. We used the random effects model to compute pooled estimations, taking into account the different sampling methods of the selected studies. To recognize any publication bias, Begg’s and Egger’s approach was performed both graphically and statistically [55, 56]. We considered the *P* value of 0.05 as statistically significant. The association between socio-demographic, clinical and service use determinants and HRS were proposed by an OR and 95% CI. We visualized the obtained results in forest plots. For data analysis, R 3.5.1 with the “meta” package was applied to perform the meta-analysis.

## Results

### Study characteristics

**Fig. 1:**
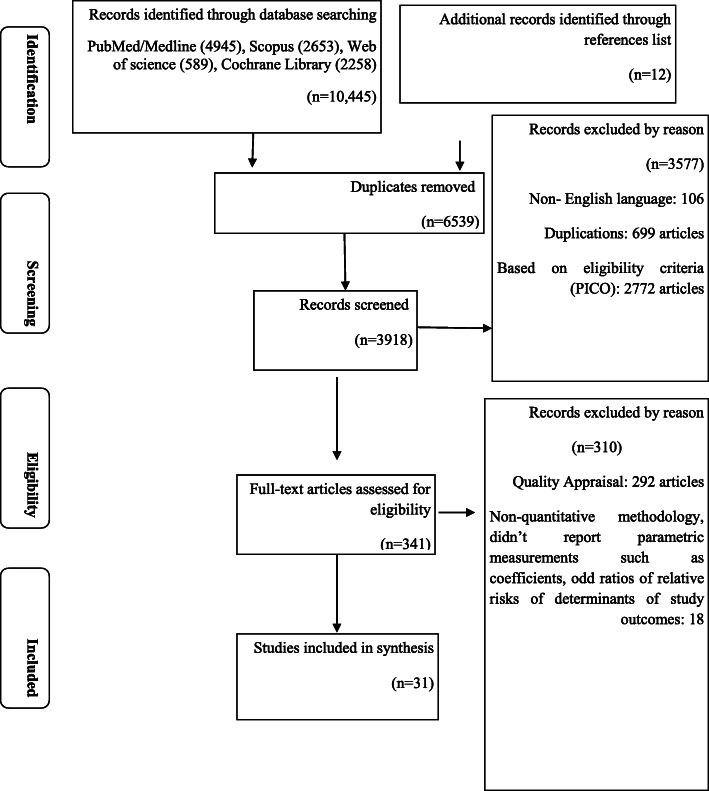
PRISMA flow diagram

The study selection process is shown in Fig. 1. There were 10,457 published papers founded in the 6 databases searched, including the article references reviewed. After article duplicates were excluded, assessments of abstracts completed and full text papers evaluated, 31 studies were retained for inclusion in this meta-analysis [9, 15, 26–54].

Out of the 31 studies, 10 were based on data collected from the Americas [*n* = 5108 participants], seven from the African Region [*n* = 24,182 participants], one from the European Region [*n* = 381 participants], three from South-East Asia [*n* = 11,112 participants] and ten from the Western Pacific Region [*n* = 17,845 participants]. China was the country with the highest number of included studies (10 studies, 11,112 participants). Considering the World Bank country income level, 9 studies (*n* = 4877) were from high-income countries, 16 studies (*n* = 37,812) from an upper middle income country, 4 studies (*n* = 15,342) were from lower middle income countries and 2 studies (*n* = 597) were from lower income countries.

### Results of the meta-analysis

We analyzed the association of socio-demographic (age > 30 years, being female, illiterate and primary education, <$USD100 monthly income, living with a spouse), clinical (CD4 count < 200,), and service use (previous HIV testing, medication adherence, poor access to care, time since diagnosis and accessibility to care) determinants for HRS among PWLH (see Table 3). These relationships were derived from information obtained from studies that recruited about 70,000 PLWH.

**Table 3:** Main characteristics of the studies selected

Author	Participants	Year of publish	Sample size	Year of implementation	Country	Design	Quality of the evidence
Charles et al. [9]	PLWHA	2012	400	2009	India	Cross-section	Good
Waite et al. [26]	PLWHA	2008	204	2001	USA	Cross-section	Satisfactory
Li et al. [27]	PLWHA	2018	239	2014	China	Cross-section	Good
Egbe et al. [28]	PLWHA	2020	385	2018	Cameron	Cross-section	Good
Emlet et al. [29]	PLWHA	2014	960	2013	Canada	Cross-section	Good
Akena et al. [30]	PLWHA	2012	368	2012	Uganda	Cross-section	Satisfactory
Letshwenyo-Maruatona et al. [31]	PLWHA	2019	4045	2013	Botswana	Cross-section	Satisfactory
Chan et al. [32]	PLWHA	2012	229	2006–2011	Uganda	Cross-section	Good
Wu et al. [33]	PLWHA	2015	940	2010–211	China	Cross-section	Very Good
Rael and Hampanda [34]	PLWHA	2015	231	2014	Mexico	Cross-section	Good
Zhang et al. [35]	PLWHA	2019	193	2018	China	Cross-section	Good
Peltzer and Ramlagan [36]	PLWHA	2011	735	2007–2008	South African	Cross-section	Very Good
Zhang et al. [37]	PLWHA	2016	2987	2012–2013	China	Cross-section	Good
Srithanaviboonchai et al. [38]	PLWHA	2017	10,522	2014	Thailand	Cross-section	Good
Li et al. [39]	PLWHA	2017	4050	2007–2008	China	Cross-section	Good
Stangl et al. [40]	PLWHA	2019	4053	2013–2015	South Africa and Zambia	Cross-section	Very Good
Stevelink et al. [41]	PLWHA	2011	190	2009	India	Cross-section	Very Good
Zhang et al. [42]	PLWHA	2016	2987	2012–2013	China	Cross-section	Very Good
Rivera et al. [43]	PLWHA	2015	716	2015	USA	Cross-section	Good
Sayles et al. [15]	PLWHA	2009	202	2007	USA	Cross-section	Satisfactory
Li et al. [44]	PLWHA	2011	202	2009	China	Cross-section	Good
Genberg et al. [45]	PLWHA	2009	14,367	2005–2006	Sub-Saharan Africa	Cross-section	Good
Earnshaw et al. [46]	PLWHA	2013	95	2013	USA	Cross-section	Very Good
Burke et al. [47]	PLWHA	2020	381	2012–2014	Russia	Cross-section	Very Good
Turan et al. [48]	PLWHA	2017	1356	2014–2015	USA	Cross-section	Good
Mao et al. [49]	PLWHA	2017	1254	2012–2013	China	Cross-section	Good
Zhang et al. [50]	PLWHA	2016	2987	2012–2013	China	Cross-section	Satisfactory
Rintamaki et al. [51]	PLWHA	2019	204	2001	USA	Cross-section	Good
Liu et al. [52]	PLWHA	2020	2006	2016	China	Cross-section	Good
Wolitski et al. [53]	PLWHA	2008	637	2008	USA	Cross-section	Good
Levi-Minzi and Surratt [54]	PLWHA	2014	503	2014	USA	Cross-section	Very Good

**Fig. 2:**
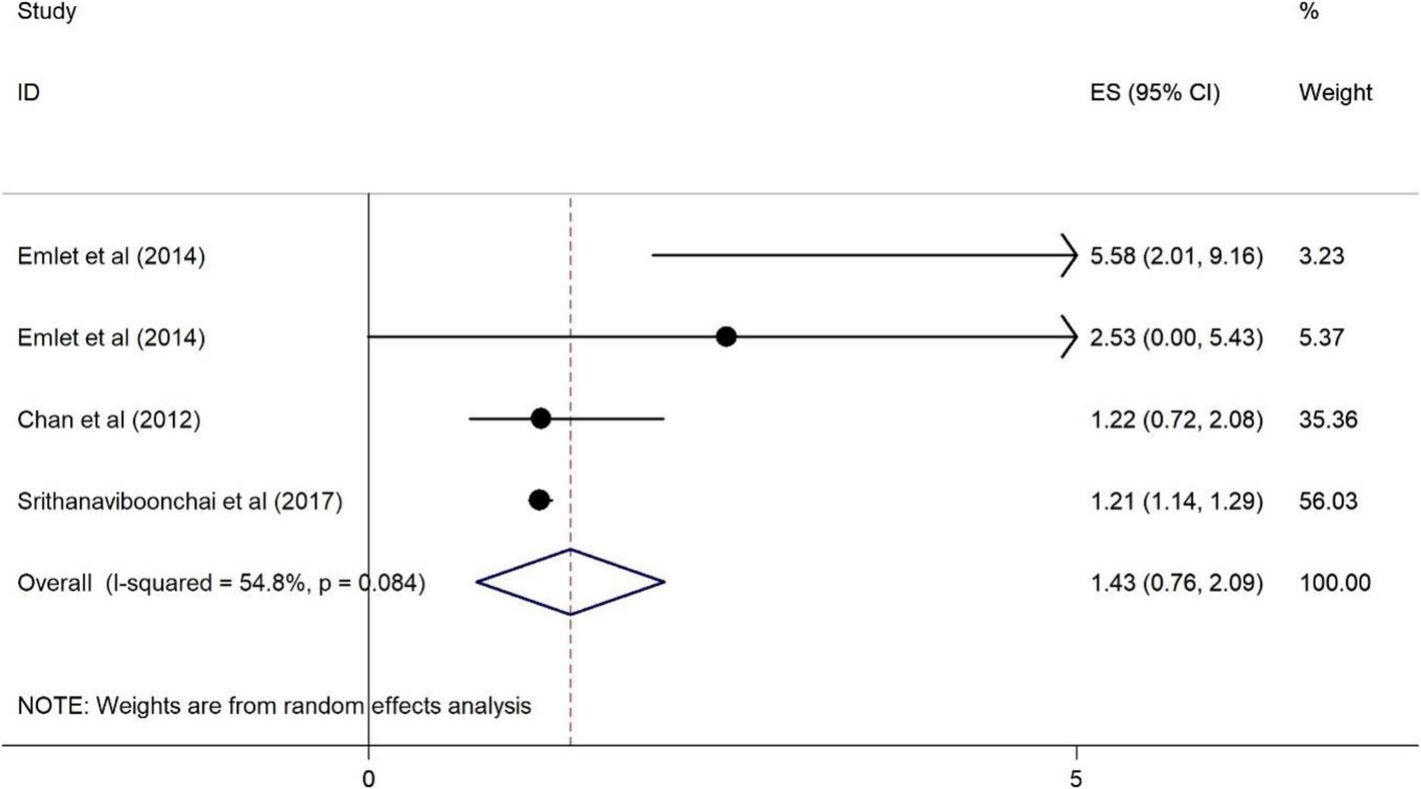
Forest plots for the association between being female and HIV related stigma among PLWH

**Fig. 3:**
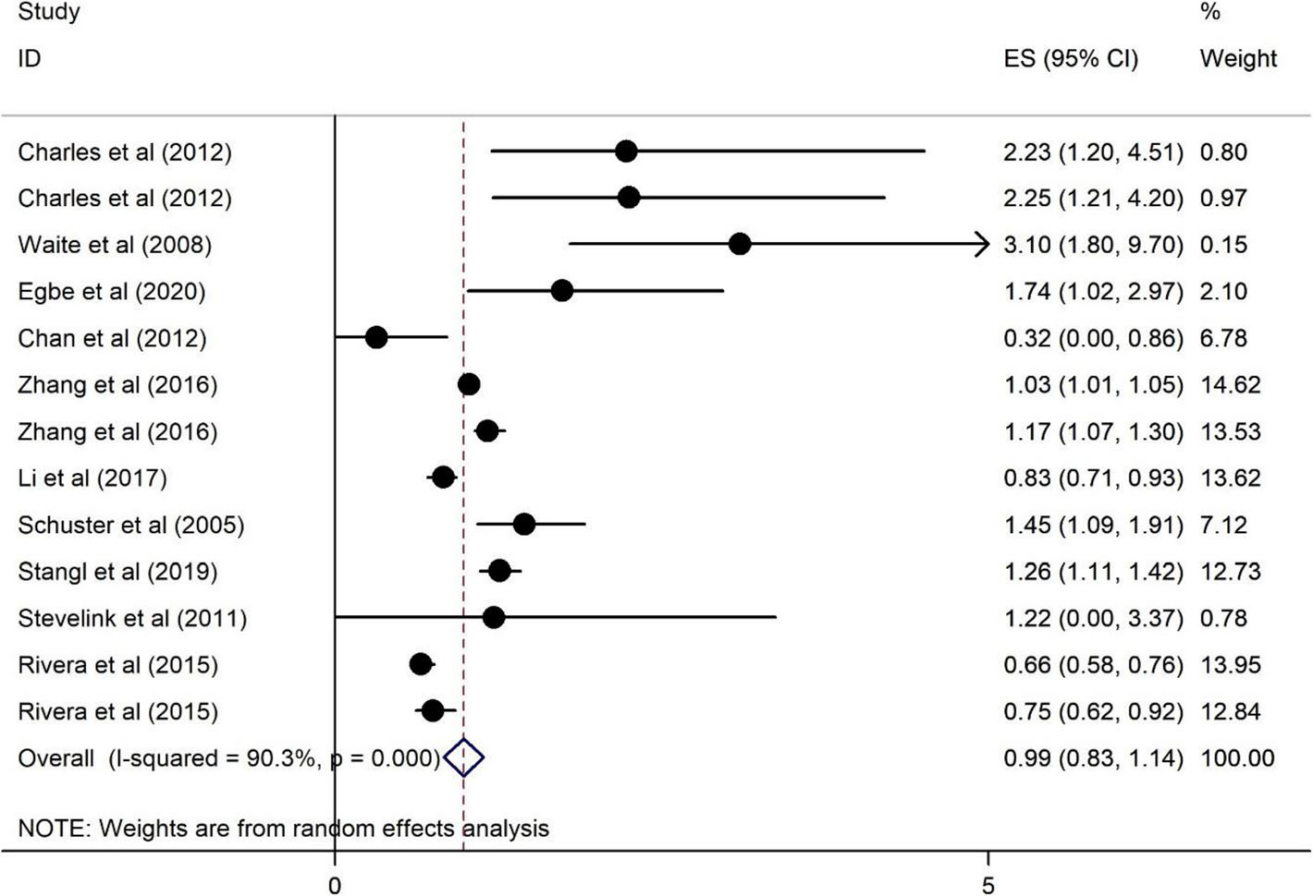
Forest plots for the association between non-literate and primary education and HIV related stigma among PLWH

**Fig. 4:**
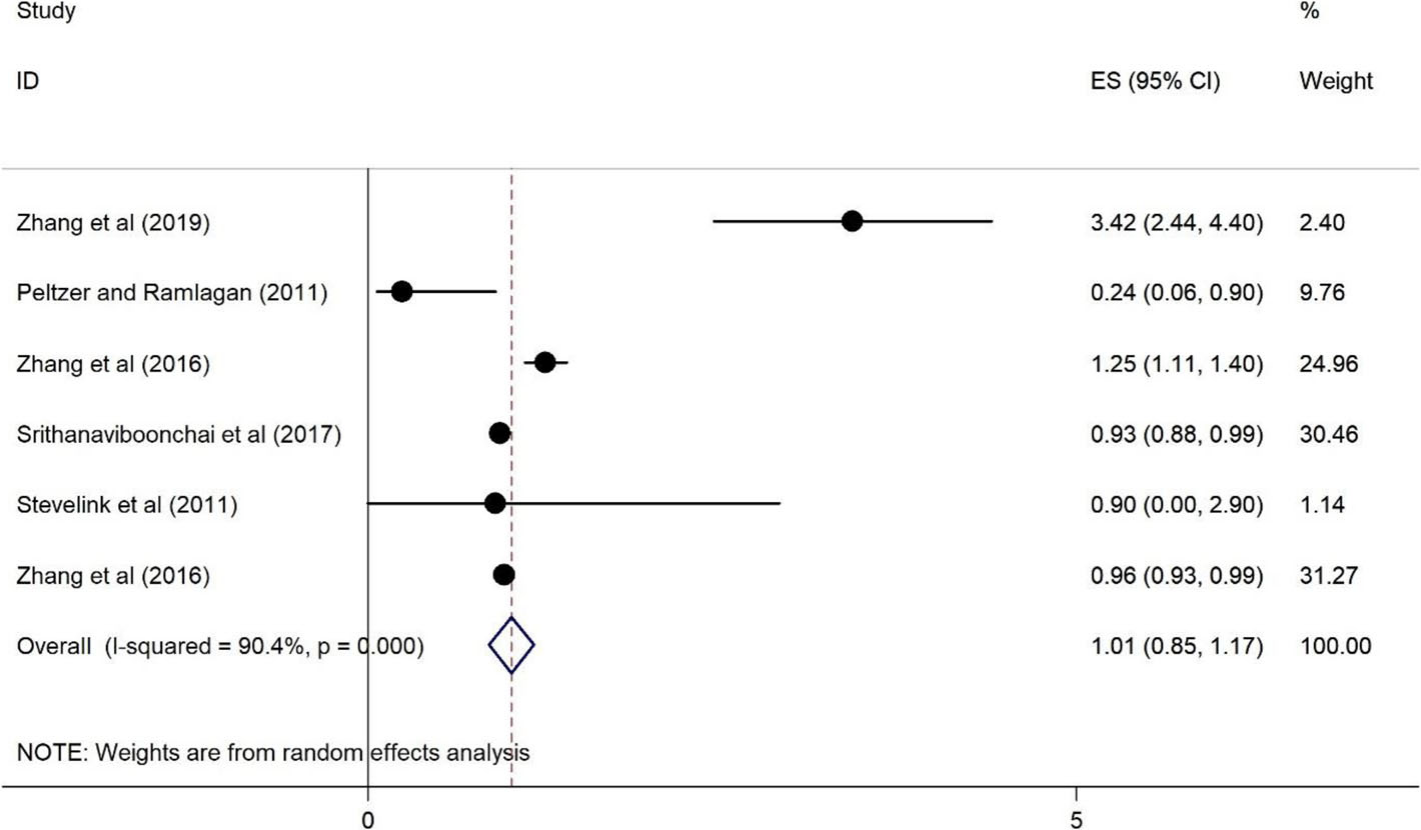
Forest plots for the association between income (< 100$) and HIV related stigma among PLWH

**Fig. 5:**
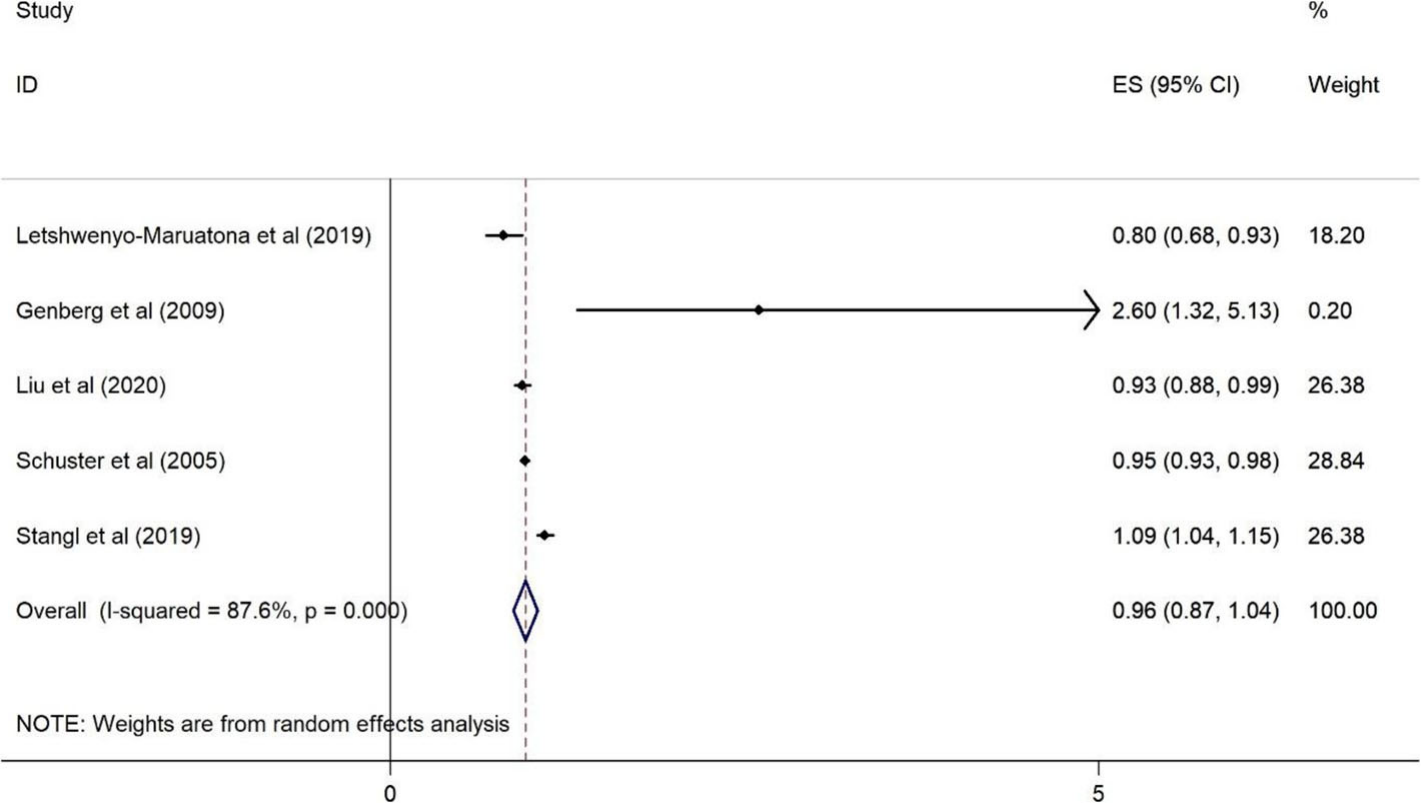
Forest plots for the association between previous HIV testing and HIV related stigma among PLWH

Plots 2 to 11 represent the associations we found. As illustrated in Figs. 2, 3, 4, 5, there were no significant associations between being female, (OR = 1.43, 95%CI = 0.76–2.09), illiterate and primary education (OR = 0.99, 95%CI = 0.83–1.14), <$USD100 monthly income (OR = 1.01, 95%CI = 0.85–1.17), and previous HIV testing and HRS (OR = 0.96, 95%CI = 0.87–1.04).

### Socio-demographic determinants

#### The association of age > 30 years on HRS among PLWH

**Fig. 6:**
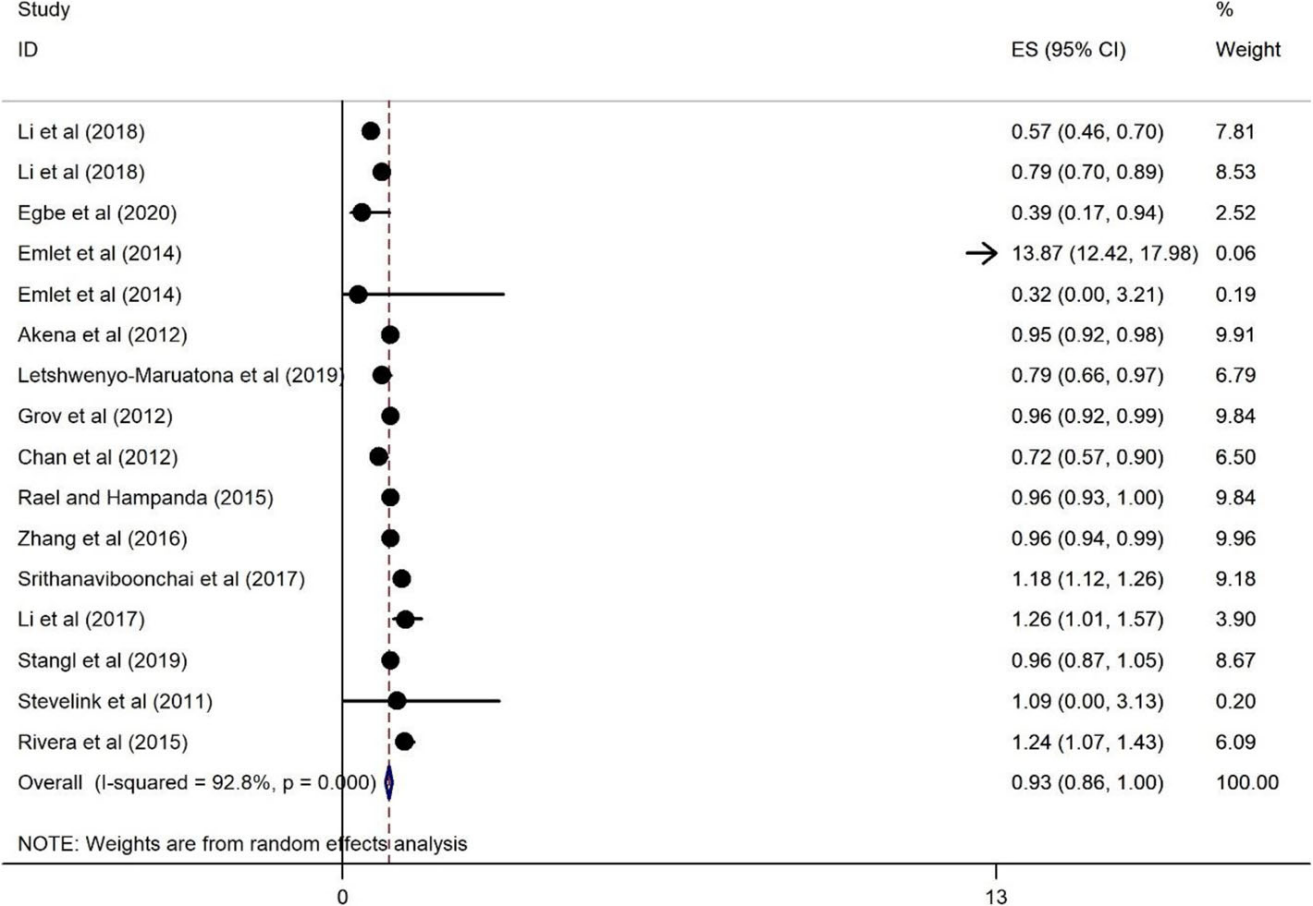
Forest plots for the association between age > 30 years and HIV related stigma among PLWH

The protective association of age > 30 years on HRS among PLWH is shown in Fig. 6 and the source of heterogeneity that has been achieved is 92.8%. The pooled effect size has a protective association and its lower bound is about 0.86 and the higher bound about 1. The OR 0.93 indicates a protective association for age > 30 years on reporting HRS (OR = 0.93, 95%CI = 0.86, 1).

#### The association of living with a spouse on HRS among PLWH

**Fig. 7:**
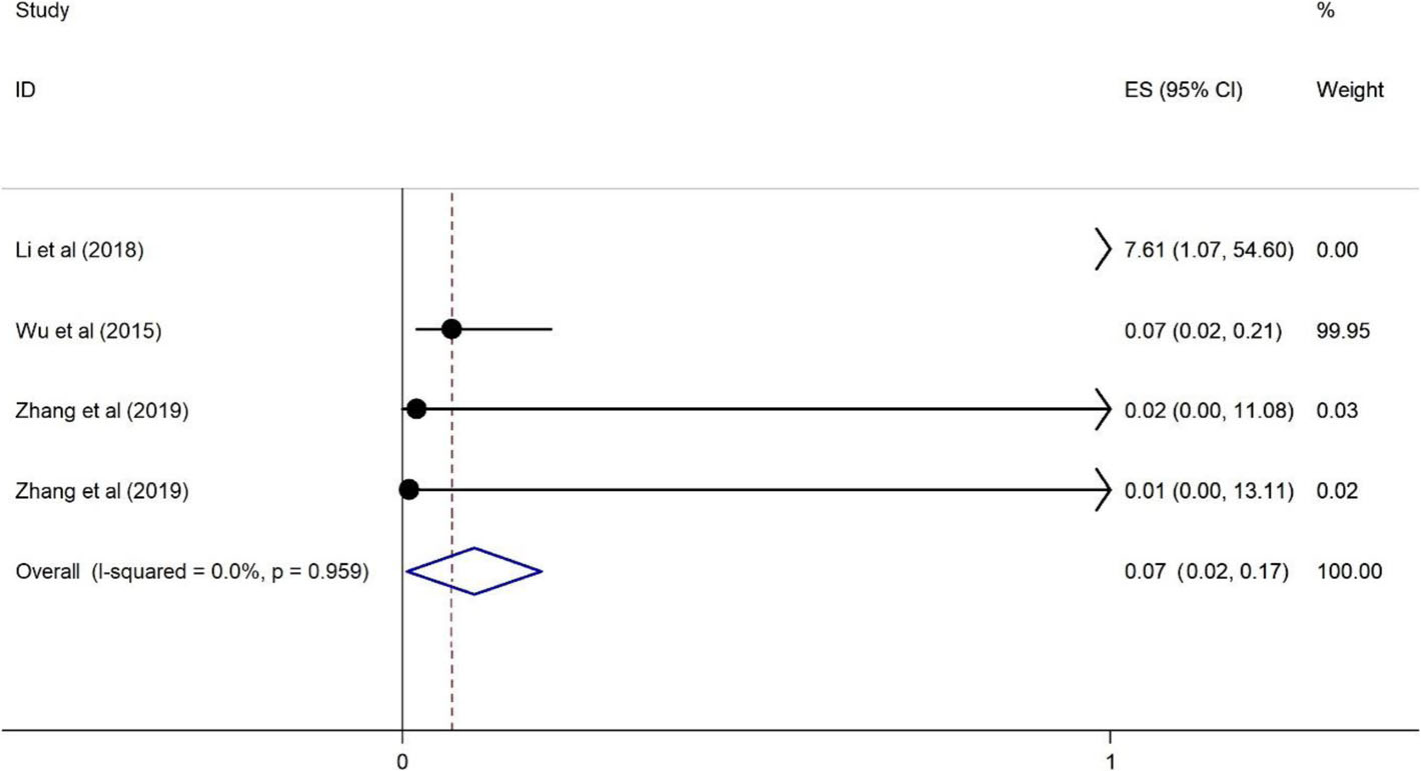
Forest plots for the association between living with spouse and HIV related stigma among PLWH

Our finding indicates a protective association between residing with a spouse and reporting HRS. PLWH living with a spouse were less likely to have reported HRS (OR = 0.07, 95%CI = 0.02, 0.17) and the heterogeneity is about 0% indicating variability in the data (See Fig. 7).

### Clinical determinants

#### The association of CD4 count < 200 on HRS among PLWH

**Fig. 8:**
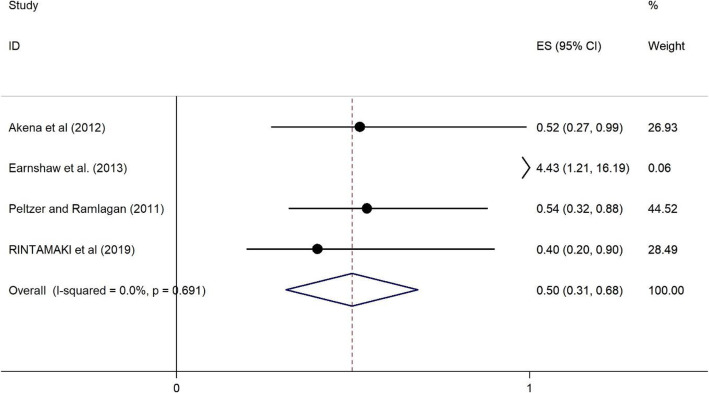
Forest plots for the association between CD4 count < 200 and HIV related stigma among PLWH

Four of the studies [30, 36, 46, 51] examined the association of CD4 count < 200 on HRS. Two studies from high-income countries [46, 51], one from a upper middle-income country [36] and one study was conducted in a low income country setting [30]. The studies were published between 2011 and 2019, and the sample sizes ranged from 95 to 735. All four studies used a cross-sectional design. The results of CD4 count on HRS among PLWH are presented in Fig. 8. There is a protective association between CD4 count (CD4 < 200) and the reporting of HRS. Those PLWH with CD4 < 200 were 0.5 times less likely to have HRS (OR = 0.5, 95%CI = 0.31, 0.68) and the heterogeneity is 0% (See Fig. 8).

### Service use determinants

#### The association of medication adherence on HRS among PLWH

Six cross-sectional studies [44, 47, 48, 50, 53, 54] examined the relationship between ART adherence and HRS. Three studies were conducted in high-income countries [48, 50, 53, 54] and published between 2008 and 2020 with sample sizes ranging from 202 to 2987.

**Fig. 9:**
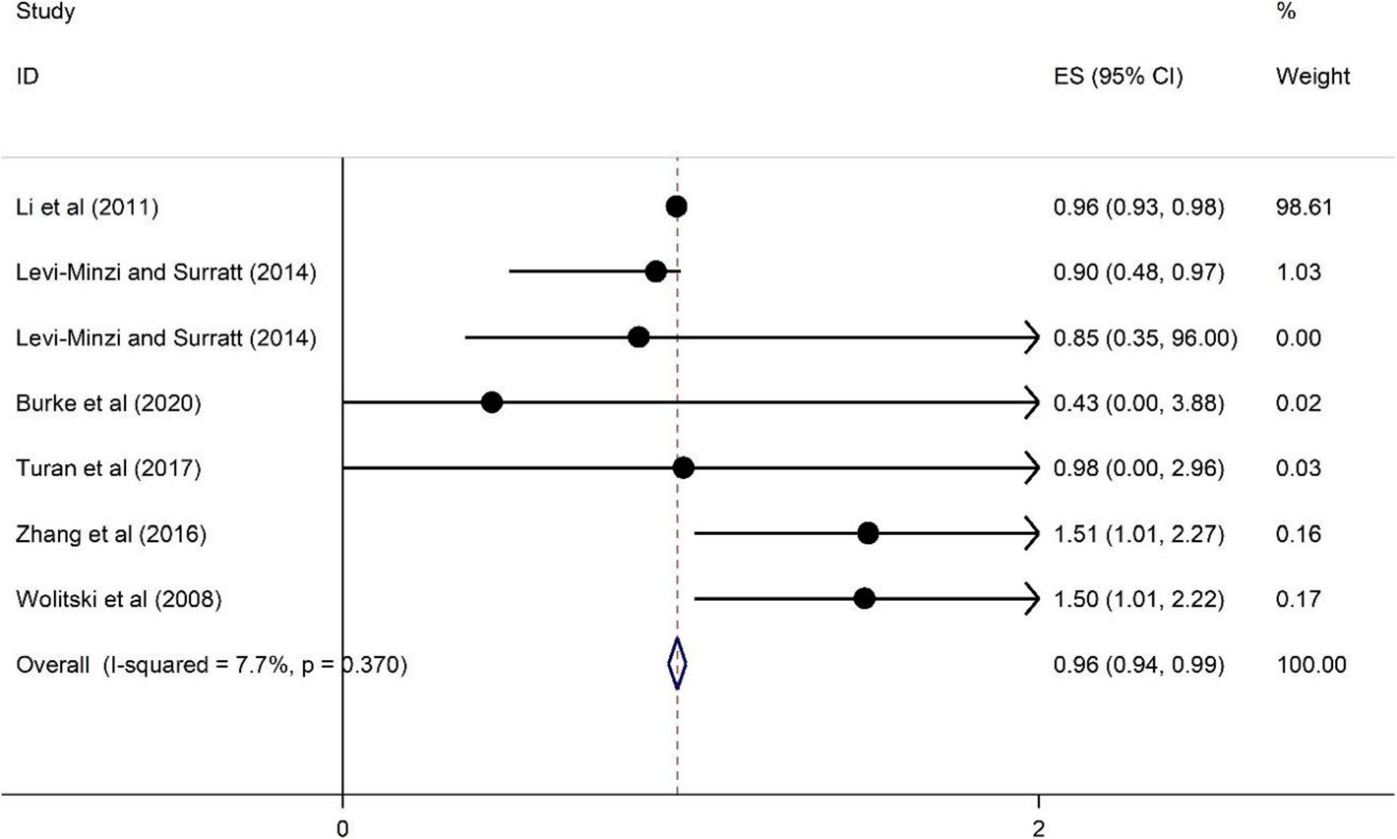
Forest plots for the association between medication adherence and HIV related stigma among PLWH

As illustrated in Fig. 9 medication adherence has a protective association on the reporting of HRS among participants. The heterogeneity statistic is about 7.7%, and the pooled effect size implies a relative neutral association. Those respondents who reported medication adherence were 0.96 times less likely to report HRS (OR = 0.96, 95%CI = 0.94, 0.99) (See Fig. 9).

#### The association of poor access to care on HRS among PLWH

**Fig. 10:**
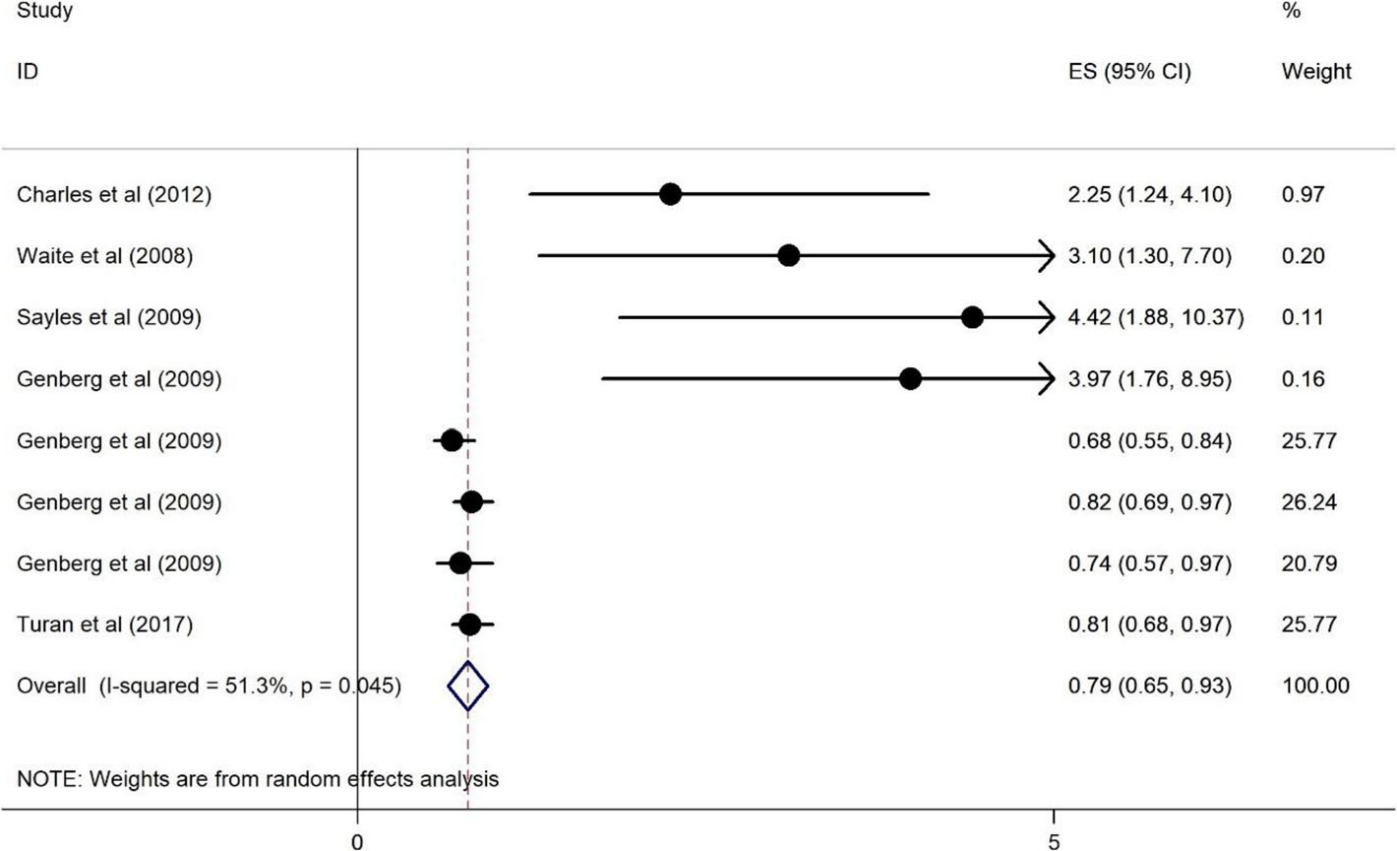
Forest plots for the association between poor access to care and HIV related stigma among PLWH

Five studies [9, 15, 26, 45, 48] examined the association of poor access to care on HRS, three from high-income countries [15, 26, 48]. One study was based in a lower middle-income country [9] and the last study was conducted in a low income country [45]. The studies were published between 2008 to 2017, and the sample sizes ranged from 202 to 14,367. All four studies used a cross –sectional design. The access to care results are presented in Fig. 10. They show a protective association between poor access to care and HRS among PLWH. Those respondents who reported poor access to care were less likely to report HRS (OR = 0.79, 95%CI = 0.65, 0.93) and the heterogeneity is 51.3% (See Fig. 10).

#### The association of time since diagnosis and accessibility to care on HRS among PLWH

Our results include three cross sectional studies that assessed the association of time since HIV diagnosis and accessibility to care on HRS. One study from an upper middle income country [49], one was from low income country [32] and one study from a high income country [29]. Studies were published between 2015 to 2017, and the sample sizes ranged from 220 to 1254.

**Fig. 11:**
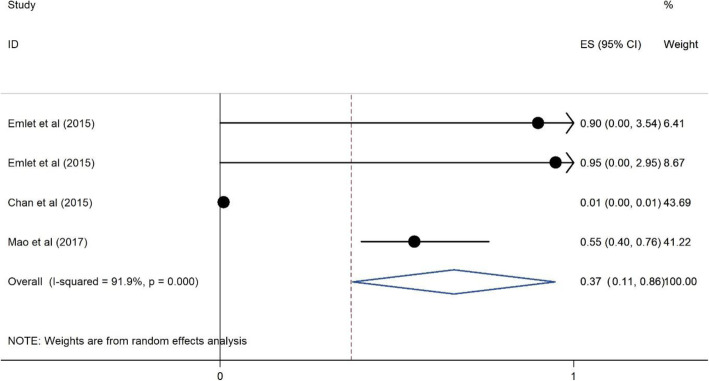
Forest plots for the association between of time diagnosis and accessibility to cares and HIV related stigma among PLWH

Figure 11 shows the protective association between time since HIV diagnosis and accessibility to hospital services and the association that has on HRS among PLWH. Those who diagnosed early were less likely to report experiencing HRS (OR = 0.37, 95%CI = 0.11, 0.86).

#### Publication bias

The publication bias was significant in the demographical subgroup including those with incomes of >$100 USD per month (C = 10.09, *P* value = 0.015) (See Table 4).

**Table 4:** Findings of publication bias using Eggers test

Demographical	Age 30	C = 3.98, *P* value = 0.179
	Being female	Not calculated
	Living with a spouse	Not calculated
	more 100 dollar	C = 10.09, *P* value = 0.015
	Non- literate and primary education	C = 1.69, *P* value = 0.173
Clinical	Poor access to care	C = 1.86, *P* value = 0.360
	Medical adhe	C = 1.28, *P* value = 0.195
	CD4	C = 1.82, *P* value = 0.576
	Previous testing	C = 3.810, *P* value = 0.502
	Time since HIV	Not calculated

## Discussion

This review explored the correlations between various socio-demographic, clinical, and service use and HRS. We examined and integrated the outcomes from 31 peer-reviewed publications published before November 2020. Significant variability was detected in terms of evaluating the socio-demographic of study participants and the reporting of HIV-related stigma. We found significant and consistent associations between reporting stigma and being aged over 30 years, living with a spouse, as socio-demographic factors and having CD4 counts of < 200, HIV medication adherence, overall poor access to care, length of time since diagnosis and accessibility to HIV care as service use.

### Assumption 1: socio-demographic characteristics have an association on HRS among PLWH

We found that living with a spouse was an important factor in reporting health related stigma. This finding is consistent with previous studies [27, 57] where living with a spouse might mediate the need to disclose their HIV status to others, therefore potentially protecting them from experiencing community based stigma.

In the present study we found that increasing age was significantly associated with lower rates of reporting HIV-related stigma. This finding is consistent with previous studies among PLWH [27, 58, 59]. In a longitudinal cohort study of almost 1000 PLWH in Ontario (Canada) it was found that while people’s experiences of stigma may not decrease with age, the authors suggest that the internalization of stigma may indeed lessen over time [29]. It may also be explained by PLWH who are older having more social and family support therefore limiting their exposure to HIV-related stigma.

However, others socioeconomic variables (being female, having low levels of education and income) do not appear to have an association on the reporting of HRS. Positive socio-demographic characteristics were more strongly associated with HIV-related stigma than negative ones.

Our meta-analysis study data could be beneficial for the development of interventions designed to reduce exposure to HIV-related stigma and focused on improving the overall mental-health health status of PLWH. Appropriate measures are required to target several populations (in terms of age, health, and socioeconomic status) for reducing various dimensions of HIV-related stigma (e.g. health care professionals, policymakers, PLWH and the communities in which they live) [16, 60, 61]. HIV-related stigma could be prevented through the implementation of structural interventions [16], and by improving the economic status of PLWH, [60]. PLWH and health care professionals could utilize the current meta-analysis data to improve awareness of the most deleterious correlates of stigma and socioeconomic status. Stigma-reduction approaches that address the socioeconomic status needs of PLWH could be designed and implemented [62–64].

### Assumption 2: clinical determinants have an association on HRS among PLWH

We found the protective correlation between HRS and CD4 counts. A previous study using multivariate data found that HIV-positive females revealed no relationship between HIV-related stigma and the physical health variables collected (i.e., viral load and CD4 counts) [65]. This highlights the importance of further research with an emphasis on controlling for potential confounding variables (e.g., sexual orientation, substance use).

We observed increased rates of HRS being reported by PLWH with lower CD4 counts. This may be explained by the effectiveness of ART medication adherence [66] it also suggests that individuals with CD4 count under 200 are likely to require more attention from health care workers including palliative and supportive care and thereby exposing them to possible stigma. Previous studies have demonstrated an association between immunological status and health related quality of life where PLWH with higher CD4-cell count reported better physical health status [67–69]. PLWH who stared their ART at CD4 < 200 cells/μl showed improved physical health status when compared to PLWH with 200–350 cells/μl or > 350 cells/μl, primarily because their physical health was worse when initiating treatment [70]. HIV treatment guidelines propose early initiation of ART (i.e. 350–500 celles/μl), as this slows the progression of HIV to AIDS and decreases mortality [71].

### Assumption 3: service use characteristics have an association on HRS among PLWH

We also detected a mediating role for HIV-related stigma on service use (i.e., adherence to ART, and access to and use of other health and social services). In line with this finding, a similar systematic review and meta-analyses demonstrated that HIV-related stigma negatively association adherence to treatment especially when social support and adaptive coping strategies are undermined [72].

PLWH who face stigma or anticipate experiencing it may try to hide their HIV infection status which in turn may lead to late initiation or interrupted ART medication [8, 72]. Some studies failed to identify any correlation between HIV-related stigma, and accessing and using health and social support [73, 74]. However, other research has found HIV-related stigma was significantly related to decreased healthcare access [10] or delay in receiving such care [75] or perceived discrimination by healthcare staff [76, 77].

A large and increasing body of literature addresses the reverse correlation between stigma and pre-exposure prophylaxis [78], HIV care adherence and retention [10, 79, 80], HIV testing [81–83], adherence to HIV medications [72, 84–86] as well as the suppression of viral load [81, 87, 88]. Previous work also suggests that HIV-based stigma negatively affects adherence to medication treatment in PLWH with most studies using a single measure of stigma to show adherence to treatment and stigma are correlated.

The present research data on the association between HIV-related stigma and reduced adherence to antiretroviral therapy is in line with prior research with specific study populations, such as injection drug users, females, and PLWH living outside the typical disease epicenters [13, 89]. Moreover, considering the above-mentioned points as well as the importance of adherence to HIV medication, stigma-centered conversations by clinicians with their patients must be considered before prescribing the patients with antiretroviral therapy. This may also involve more discrete pharmacotherapy regimens for PLWH highly sensitive to stigma as well as psychoeducational programs that increase social support and improve resilience. Further research is necessary to explore the association of community- and clinical-based stigma on the mental health needs of PLWH. Such measures could help eliminate the impact of stigma and reveal the influence of mediating factors on the HIV-induced stigma. Such data could be beneficial when developing HIV care and management plans that support PLWH.

Our findings are in line with those of previous research which highlights the impact of HRS on ART adherence, specifically in resource-limited settings [90, 91]. Our data could be used by policymakers to develop and implement HIV focused public health policies, including universal voluntary testing with immediate treatment in high prevalence HIV populations [92].

Similarly to results from Southern Wollo, Ethiopia [75] our study found stigma and HIV were related to late diagnosis of disease, i.e. participants reporting high levels of stigma were more likely to present late for HIV testing and diagnosis. This may be explained by internalized stigma, where those who remain un-tested do so because they do not want to face the stigma they perceive will be evident when or if they test positive to HIV.

The present finding is also in line with data from HIV positive African Americans [93] where HIV-related stigma was found to decrease over time as those living with HIV receive information and come to more comprehensively understand their disease. In doing so they find mental health and social support to deal effectively with HIV stigma.

### Strength and limitation of study

Our study had some limitations should be acknowledging, this is the first quantitative literature synthesis on the relationship between HIV-related stigma and the demographic, clinical and service use variables of PLWH. By restricting our study to peer-reviewed journals the data might be subject to publication bias. As we only included observational studies; we are unable to infer causal relationships between reported HIV-related stigma and other characteristics. The studies included applied a variety of instruments for assessing HIV-related stigma and several studies developed and applied bespoke data collection tools or analyzed self-reported data. The limited number of studies (*N* = 31) also precluded subgroup analyses. Extending our understanding of the factors associated with HIV-related stigma could be beneficial for healthcare professionals working with PLWH and their support networks. Additionally, our study did not include research regarding the impact of stigma on pre-ART correlation with treatment [94, 95], or in people who refuse ART [160].

### Implications for future intervention design

The relationship between the social and clinical determinants and HRS and adherence to ART is important when designing future interventions to enhance and improve adherence. For example, correlations between internalized stigma, and adherence need interventions that includes a cognitive-behavioral component (e.g., challenging internalized opinions related to affecting HIV, improving motivation to preserve optimal health,).

## Conclusions

Our study supports the idea that HIV-related stigma has an association with a variety of health- related outcomes in people who are living with HIV. Our research demonstrated significant associations between HIV-related stigma and being aged > 30 years, living with a spouse, CD4 count < 200, adherence to antiretroviral therapy, and service availability. Our findings suggest that health care professionals and others working with PLWH can also assist in the planning of strategies which decrease the stigma related with taking medication. Our findings may be useful for PLWH and those working with them to improve knowledge of the most deleterious associations of stigma with physical and mental health. Our data suggest interventions that address stigma must focus on age, socio-economic status, gender, and culture and should be designed to identify experiences of social stigma, to reduce the most negative aspects of physical/mental health for PLWH. This may ultimately help to create environments that facilitate social support groups for PLWH and the broader community. Considering the protective association stigma has on HIV prevention and treatment for PLWH, it is recommended that targeted evidence-based HIV stigma reduction interventions be developed. Focusing rigorously designed and evaluated interventions on a particular group, such as healthcare professionals and applying may also help to reduce HRS.

## Data Availability

The datasets used and/or analyzed during the current study are available from the corresponding author on reasonable request.

## Abbreviations

HRS: HIV-related stigma
PLWH: People living with HIV
PICO: Population, Intervention, Comparator, Outcomes
OR: Pooled odds ratio
ART: Antiretroviral therapy
PRISMA: Protocols of Systematic Reviews and Meta-Analyses
WHO: World Health Organization
